# Vascular burden and genetic risk in association with cognitive performance and dementia in a population-based study

**DOI:** 10.1016/j.cccb.2022.100145

**Published:** 2022-05-05

**Authors:** Marios K. Georgakis, Eva Ntanasi, Alfredo Ramirez, Benjamin Grenier-Boley, Jean-Charles Lambert, Paraskevi Sakka, Mary Yannakoulia, Mary H. Kosmidis, Efthimios Dardiotis, Georgios M. Hadjigeorgiou, Sokratis Charissis, Niki Mourtzi, Alexandros Hatzimanolis, Nikolaos Scarmeas

**Affiliations:** a Institute for Stroke and Dementia Research (ISD), University Hospital, Ludwig-Maximilians-University LMU, Feodor-Lynen-Str. 17, Munich 81377, Germany; b Center for Genomic Medicine, Massachusetts General Hospital, Boston, MA, USA; c Programme in Medical and Population Genetics, Broad Institute of MIT and Harvard, Cambridge, MA, USA; d 1st Department of Neurology, Eginition Hospital, National and Kapodistrian University of Athens Medical School, Athens, Greece; e Department of Nutrition and Dietetics, Harokopio University, Athens, Greece; f Department of Psychiatry, Medical Faculty, University of Cologne, Cologne, Germany; g Department of Neurodegenerative Diseases and Geriatric Psychiatry, University of Bonn, Bonn, Germany; h U1167-RID-AGE Facteurs de Risque et Déterminants Moléculaires des Maladies Liés au Vieillissement, University of Lille, Inserm, CHU Lille, Institut Pasteur de Lille, Lille, France; i Athens Association of Alzheimer's Disease and Related Disorders, Marousi, Greece; j Lab of Cognitive Neuroscience, School of Psychology, Aristotle University of Thessaloniki, Thessaloniki, Greece; k Department of Neurology, Faculty of Medicine, School of Health Sciences, University Hospital of Larissa, University of Thessaly, Larissa, Greece; l Department of Neurology, Medical School, University of Cyprus, Nicosia, Cyprus; m Department of Psychiatry, National and Kapodistrian University of Athens Medical School, Eginition Hospital, Athens, Greece; n Theodor-Theohari Cozzika Foundation, Neurobiology Research Institute, Athens, Greece; o Department of Neurology, The Gertrude H. Sergievsky Center, Taub Institute for Research in Alzheimer's Disease and the Aging Brain, Columbia University, New York, NY, USA

**Keywords:** Genetics, Cardiovascular prevention, Vascular risk factors, Dementia, Cognitive decline, Population-based studies

## Abstract

**Background and purpose:**

Vascular risk factors may influence cognitive function and thus represent possible targets for preventive approaches against dementia. Yet it remains unknown, if they associate with cognition independently of the individual genetic risk for dementia.

**Methods:**

In a population-based study of 1172 community-dwelling individuals aged ≥65 years in Greece, we constructed a vascular burden score (VBS; based on presence of hypertension, diabetes, hyperlipidemia, heart disease, and cerebrovascular disease, range 0–5) and a polygenic risk score (PRS) for clinically-diagnosed Alzheimer's disease (AD) based on 23 genetic variants. We then explored in joint models the associations of the PRS for AD and VBS with global cognitive performance, cognitive performance across multiple cognitive domains, and odds of dementia.

**Results:**

The mean age of study participants was 73.9 ± 5.2 years (57.1% females). Both the PRS for AD and VBS were associated with worse global cognitive performance (beta per-SD-increment in PRS: -0.06, 95%CI: -0.10 to -0.02, beta per-point-increment in VBS: -0.05, 95%CI: -0.09 to -0.02), worse performance across individual cognitive domains (memory, executive function, attention, language, visuospatial ability), and higher odds of dementia (OR per-SD increment in PRS: 1.56, 95%CI: 1.17–2.09, OR per-point increment in VBS: 1.38, 95%CI: 1.05–1.81). There was no evidence of an interaction between the two scores. Higher VBS was associated with worse cognitive performance equally across tertiles of the PRS for AD, even among individuals at the highest tertile.

**Conclusions:**

Both genetic risk and vascular burden are independently and additively associated with worse cognitive performance and higher odds of dementia.

## Highlights

• A vascular burden score and a genetic risk score for dementia were both associated with higher risk of cognitive impairment and dementia.
• There was no interaction between the genetic score and the vascular burden score in their associations with cognitive impairment or dementia.
• Even among individuals with high genetic score, a low vascular burden score was associated with better cognitive performance.
• In a community-based sample, vascular risk and genetic risk are associated with cognitive performance and odds of dementia additively and independently of each other.
• Whether targeting vascular risk factors could offset a high genetic risk score for dementia should be further explored in future studies.

## Introduction

Dementia is a devastating clinical diagnosis posing a substantial burden on patients, their proxies, and public healthcare systems [1,2]. Given the lack of available treatments for deceleration or regression of cognitive decline, developing effective preventive strategies against cognitive impairment is crucial. Towards this goal, it is important to elucidate the etiology of cognitive impairment in later life. A number of population-based studies have provided evidence about how vascular risk factors associate with the risk of cognitive impairment and dementia in later life [3], [4], [5], [6], [7]. Individuals with a higher burden of vascular risk factors and vascular disease show a rapider cognitive decline, faster progression to dementia, and accelerated brain atrophy [8], [9], [10]. As vascular risk factors and vascular diseases are potentially modifiable, they have received great attention as potential approaches for prevention of cognitive decline in later life [11].

Yet, moving towards personalized preventive approaches would require considering the individual background risk for dementia. Recent large-scale meta-analyses of genome-wide association studies (GWAS) have provided important insights with regards to genetic factors increasing the risk of Alzheimer's disease, which underlies 70% of all dementia cases [12,13]. Up to 29 genomic loci have been to date identified as increasing the risk of Alzheimer's disease [12]. Combining genetic variants in these loci into a polygenic risk score (PRS) could provide important insights about the individual genetic risk for dementia [14]. Indeed, such PRSs predict cognitive decline, the risk of developing mild cognitive impairment and dementia among cognitively normal individuals, as well as the risk of conversion to dementia among individuals with mild cognitive impairment [15,16]. It still remains unclear though whether there is any interaction between the background genetic risk for dementia and the vascular burden score, and thus whether preventive strategies targeting vascular risk factors could offset cognitive decline even among individuals at high genetic risk for dementia.

Here, to address this issue, we use data from a population-based study of 1431 community-dwelling individuals in Greece to explore (i) whether a PRS for Alzheimer's disease is associated with cognitive performance and odds of dementia, (ii) whether a score representing the burden of vascular risk factors and vascular diseases (vascular burden score, VBS) is associated with cognitive performance and odds of dementia, (iii) whether vascular burden and genetic risk for dementia are jointly associated with cognitive performance and odds of dementia, and (iv) whether vascular burden associates with cognitive performance even among individuals with a high genetic risk for Alzheimer's disease.

## Methods

### Study population

Participants for the current study were drawn from the Hellenic Longitudinal Investigation of Aging and Diet (HELIAD) cohort. HELIAD is a population-based, multidisciplinary, collaborative study in Greece. Details about the study design and methodology are detailed elsewhere [17], [18], [19], [20], [21]. Briefly, recruitment for the study has been carried out in two different centers, one located in Marousi, a suburb of Athens and one in the city of Larissa, in central Greece, between 2011 and 2014. Participants were selected through random sampling of community-dwelling individuals of 65 years of age or older. The study has been approved by the Ethics Review Boards of the National and Kapodistrian University of Athens and University of Thessaly. All participants have given written informed consent prior to their participation.

### Data collection

In structured standardized face-to-face intensive interviews, study participants provided information regarding their medical history including previous or current diseases, neurological conditions, neuropsychiatric symptoms, current medications, hospitalizations, surgeries and injuries. Medical records of previous diagnoses, physician visits, or hospitalizations were inspected for all participants. Additionally, an extensive structured and standardized physical examination was conducted, evaluating neurological signs and symptoms. Structured questionnaires were used in order to gather information about participants’ functioning, social, mental and physical activities, as well as sleep and dietary habits. Sociodemographic information and information about tobacco use was also collected. Height and weight were measured and body mass index (BMI) was calculated.

### Neuropsychological evaluation

The evaluation of cognitive function was performed by neuropsychologists through a comprehensive neuropsychological assessment of all major cognitive domains: (i) orientation (MMSE [22]), non-verbal and verbal memory (medical college of Georgia complex figure test (MCG) [23]; Greek verbal learning test [24]); (ii) language (semantic and phonological verbal fluency [25]; subtests of the Greek version of the Boston diagnostic aphasia examination short form, namely, the Boston naming test-short form, and selected items from the complex ideational material subtest, to assess verbal comprehension and repetition of words and phrases [26]); (iii) visuoperceptual ability (Judgment of Line Orientation [27,28] abbreviated form; MCG complex figure test copy condition [29]; clock drawing test [30]); (iv) attention and information processing speed (trail making test (TMT) Part A [31]); (v) executive functioning (TMT-Part B; verbal fluency; anomalous sentence repetition; graphical sequence test; motor programming [29]; months forwards and backwards), and (vi) a gross estimate of intellectual level (a Greek multiple choice vocabulary test [32]).

Participants’ raw scores on each cognitive test were converted into *z*-scores using mean and SD values derived from the subset of cognitively normal study participants (no mild cognitive impairment or dementia). Subsequently, these individual neuropsychological test scores were used to produce an average domain composite *z*-score for memory, executive function, attention, language, and visuospatial ability [33]. The domain-specific composite *z*-scores were then averaged in order to calculate a global neuropsychological *z*-score [17]. A higher score indicates better performance. Global cognitive performance was our primary outcome, whereas domain-specific performance was a secondary outcome.

Presence of dementia was also a secondary outcome. Diagnoses of dementia were made according to the DSM-IV-TR criteria [34] in diagnostic consensus meetings including all the researchers and main investigators involved in the project, both neurologists and neuropsychologists. Changes in performance of daily activities and self-care habits that require physical capacity and cognitive functioning, in particular memory, comprehension, calculations and visuospatial orientation, were measured using the Blessed Dementia Scale [35]. The participants' ability to use the telephone and transportation, medication management and handling of finances independently was assessed using the Lawton Instrumental Activities of Daily Living scale (IADL) [36]. More details regarding the diagnostic procedures followed can be found in previously published work [19,37].

### Vascular burden score

A score reflecting the burden of vascular risk factors and vascular disease (VBS) was constructed in accordance with previous studies [9,38]. The presence and number of vascular risk factors for each participant was based on a thorough review of the participant's medical history, medical records, and medication plan. The VBS for each participant was calculated as the sum of five vascular risk factors and vascular diseases including (i) hypertension (personal history, medical records, receipt of antihypertensive medications), (ii) diabetes mellitus (personal history, medical records, or receipt of glucose-lowering medication), (iii) hyperlipidemia (personal history, medical records, receipt of lipid-lowering medications), (iv) heart disease (history of ischemic heart disease, myocardial infarction, coronary angioplasty, coronary artery bypass surgery, congestive heart failure, atrial fibrillation or other arrhythmias, implantation of a pacemaker), and (v) cerebrovascular disease (history of stroke or transient ischemic attack). For stroke and transient ischemic attacks, beyond the standard interview, participants were also interviewed for a history of neurological symptoms consistent with stroke or transient ischemic attack. When there was a history of symptoms suggestive of cerebral ischemia, further information regarding the date of onset, duration, constellation of symptoms, admission to hospital or any kind of rehabilitation treatment was elicited [39]. Presence of each of these risk factors or disease was rated with 1. As such, the final score ranged from 0 to 5.

### Genotyping in HELIAD

Genome-wide genotyping was performed at the facilities of the “Center national de recherche en génétique humaine” (Evry, France) using the Illumina Infinium Global Screening Array (GSA, GSAsharedCUSTOM_24+v1.0), as detailed elsewhere [40]. Briefly, variants included in the marker list for removal by Illumina were excluded and only variants for which the full-length probes aligned uniquely on the GRCh38 genome without any mismatch were kept. Variant intensity quality control (QC), was conducted for all autosomal variants, according to established methods [41]. Sample quality control was performed using PLINK v1.9 [42]. Samples with missingness >0.05, sex inconsistencies or with heterozygosity rate that deviated more than ±6 SD from the mean, were excluded. To identify population outliers, a Principal Component Analysis (PCA), was run using as reference the 1000 Genomes phase 3 population and the dataset was projected onto two dimensions, using the flashPCA2 software [43]. To control for cryptic relatedness, we kept one individual from each pair of samples with a kinship coefficient more than 0.125 (cut-off for second-degree relatives), yielding a final sample size of 1251 unrelated individuals. We excluded variants with a missingness>0.05 in at least one genotyping center or with a differential missingness test *p* < 10^−10^. The Hardy-Weinberg equilibrium tests (*p* < 5 × 10^–8^) were performed only in controls and for each genotyping center/country separately.

To improve the accuracy of imputation, we compared the frequencies of variants (chi-square test) against two reference panels, the population of the Haplotype Reference Consortium r1.1 (HRC) [44] excluding 1000 Genomes samples and the Finnish and non-Finnish population of Genome Aggregation Database v3 (gnomAD) [45]. Variants showing a χ^2^ >3000 in both the HRC and the gnomAD or a χ^2^>3000 in one reference panel without being present in the other were excluded. Finally, GWAS analyses were performed between controls across genotyping centers to assess frequency differences between genotyping centers, using the software SNPTEST [46], under an additive model and adjusting on associated PCs. Variants with a Likelihood Ratio Test of *p* < 10^−5^ were excluded. Furthermore, we removed ambiguous variants with minor allele frequency (MAF) > 0.4 and we kept only one copy of any duplicated variants, prioritizing the one with the lowest missingness. All samples and variants, passing the above QC metrics were imputed in the Michigan Imputation Server (v1.2.4) [47] using the TOPMed Freeze 5 reference panel. Phasing and imputation were performed using EAGLE v2.4 [48] and Minimac4 v4–1.0.2 software, respectively.

### Construction of genetic risk score for Alzheimer's disease

Imputed dosages for a total of 5611,082 SNPs with MAF>0.05, call rate >95% and imputation quality score >0.4 were converted to best-guess genotypes for PRS computation. The PRSice software (http://prsice.info/) was utilized to construct PRSs for each individual applying the clumping and thresholding (C+T) method [49]. A PRS for Alzheimer's disease was computed, as the weighted sum of the risk increasing alleles that each individual carries at each SNP locus multiplied by the effect size for the reference allele on the basis of large GWAS meta-analysis summary data (i.e. discovery samples) [12]. A set of 23 out of 29 SNPs, which reached genome-wide significance in the original GWAS meta-analysis and were available in our dataset, were used to construct the PRS for Alzheimer's disease (**Supplementary Table 1**) [12].

### Statistical analysis

First, we explored whether participant characteristics differed between individuals across tertiles of the PRS for Alzheimer's disease using two-way ANOVA, the Kruskal-Wallis test, or chi-square, as appropriately. To explore the associations of VBS and dementia PRS with cognitive performance, we designed linear regression models including age, sex, years of education, the participants’ VBS and PRS for Alzheimer's disease, as well as the first two principal components of ancestry. To explore whether the VBS and the PRS for Alzheimer's disease exert independent effects, we included both of them in the same models. Beyond global cognitive performance, which was our primary outcome, we also explored associations with cognitive performance across the 5 main cognitive domains (memory, executive function, attention, language, visuospatial ability). We also explored the same associations with odds of dementia in logistic regression models including the same variables.

We then explored interactions between the VBS and the PRS for Alzheimer's disease. For the continuous outcome of cognitive performance, we included the product of the two variables in a linear model and used its coefficient as a measure of additive interaction. For the binary outcome of dementia, we included the interaction term of the two variables in a logistic regression model and used its coefficient as a measure of multiplicative interaction. To assess the interaction on the additive scale, we calculated the relative excess risk due to interaction (RERI); confidence intervals for RERI were calculated using the delta method. We also split the sample in three tertiles depending on participants’ PRS for Alzheimer's disease and explored associations between of the VBS with global and domain-specific cognitive performance across the tertiles., All analyses were performed using R (v3.6.3; The R Foundation for Statistical Computing).

## Results

A total of 1172 individuals with available genetic and cognitive data were included in this analysis (73.9 ± 5.2 years of age, 57.1% females, 6.8 ± 4.5 years of education). The study participants across the three tertiles of a PRS for Alzheimer's disease did not differ with regards to demographic characteristics or individual vascular risk factors (Table 1). There was evidence of a modest association between the composite VBS and the PRS for Alzheimer's disease (**Supplementary Fig. 1**). There were considerable differences with regards to cognitive performance across the three tertiles of PRS for Alzheimer's disease with individuals with a higher PRS performing lower across cognitive domains (Table 1).

**Table 1:** Baseline study characteristics by tertiles of the polygenic risk score for Alzheimer's disease.

	**PRS for Alzheimer's disease**			
**Variable**	**1st tertile (*n*** **=** **390)**	**2nd tertile (*n*** **=** **390)**	**3rd tertile (*n*** **=** **392)**	***p*-value**
Age, y, mean (SD)	73.6 (5.1)	74.2 (5.3)	73.9 (5.3)	0.355
Females, N (%)	222 (56.9)	224 (57.4)	223 (56.9)	0.985
Education, y, mean (SD)	6.9 (4.5)	6.8 (4.3)	6.9 (4.7)	0.967
Hypertension, N (%)	265 (69.2)	260 (67.7)	269 (69.9)	0.803
Diabetes mellitus, N (%)	62 (16.3)	66 (17.2)	84 (21.8)	0.104
Dyslipidemia, N (%)	158 (41.2)	156 (40.6)	176 (45.7)	0.298
Cerebrovascular disease, N (%)	27 (7.0)	40 (10.4)	43 (11.1)	0.119
Myocardial infarction, N (%)	15 (3.9)	14 (3.6)	14 (3.7)	0.971
BMI, kg/m^2^, mean (SD)	29.3 (5.1)	29.3 (5.3)	29.1 (5.3)	0.771
Smoking status				0.316
Current smokers, N (%)	40 (10.2)	30 (7.7)	44 (11.2)	
Former smoker, N (%)	99 (25.4)	113 (29.0)	94 (24.0)	
Never smokers, N (%)	251 (64.4)	247 (63.3)	254 (64.8)	
Vascular burden score, median [Q1-Q3]	1 [1–2]	2 [1–2]	2 [1–2]	0.048
Cognitive performance				
Global cognitive score, mean (SD)	−0.28 (0.81)	−0.41 (0.89)	−0.44 (0.90)	0.003
Memory, mean (SD)	−0.24 (0.88)	−0.42 (0.90)	−0.37 (0.90)	0.015
Executive function, mean (SD)	−0.27 (0.80)	−0.41 (0.89)	−0.39 (0.88)	0.043
Attention, mean (SD)	−0.33 (1.18)	−0.43 (1.23)	−0.47 (1.29)	0.313
Language, mean (SD)	−0.29 (0.90)	−0.40 (0.97)	−0.36 (0.95)	0.297
Visuospatial ability, mean (SD)	−0.19 (1.02)	−0.36 (1.17)	−0.28 (1.23)	0.152
Dementia, N (%)	11 (2.8)	16 (4.1)	20 (5.1)	0.171

PRS: polygenic risk score, SD: standard deviation.

**Fig. 1:**
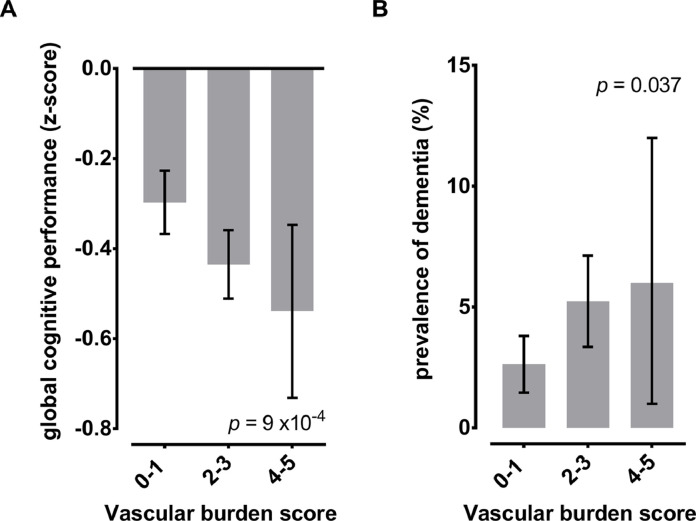
Global cognitive performance and prevalence of dementia across the range of the vascular burden score.

Individuals with a higher vascular burden, as indicated by higher scores in the VBS, also scored worse on cognitive performance and were more likely to be diagnosed with dementia (Fig. 1). In multivariable analyses, both the PRS for Alzheimer's disease (beta per 1-SD increment: −0.06, 95%CI: −0.10 to −0.02), as well as the VBS (beta per 1-point increment: −0.05, 95%CI: −0.09 to −0.02), were independently associated with worse global cognitive performance (Table 2). Similar associations were observed across all cognitive domains. Furthermore, both the PRS for Alzheimer's disease (OR per 1-SD increment: 1.56, 95%CI: 1.17–2.09) and the VBS (OR per 1-point increment: 1.38, 95%CI: 1.05–1.81) were associated with higher odds of dementia (Table 2). For all of these associations, there was no evidence of significant interactions between the PRS for Alzheimer's disease and the VBS (Table 2**)**. There was also no evidence of significant deviation from the additive scale in the associations with the odds of dementia (RERI: 0.15, 95%CI: −0.25 to 0.55; Table 2).

**Table 2:** Associations between the polygenic risk score for Alzheimer's disease and vascular burden score with cognitive outcomes from joint models including both variables.

**Outcome**	Exposure*	**Association estimate**	***p*-value**
**Cognitive performance**		**beta (95%CI)**	
Global cognitive function	PRS	−0.06 (−0.10 to −0.02)	0.002
	VBS	−0.05 (−0.09 to −0.02)	0.003
	Interaction		0.861
Memory	PRS	−0.07 (−0.12 to −0.03)	0.002
	VBS	−0.02 (−0.05 to 0.02)	0.409
	Interaction		
Executive function	PRS	−0.04 (−0.08 to −0.001)	0.048
	VBS	−0.04 (−0.08 to −0.01)	0.034
	Interaction		0.972
Attention	PRS	−0.10 (−0.16 to −0.03)	0.003
	VBS	−0.09 (−0.15 to −0.03)	0.003
	Interaction		0.927
Language	PRS	−0.04 (−0.08 to 0.00)	0.077
	VBS	−0.05 (−0.08 to −0.01)	0.016
	Interaction		0.851
Visuospatial ability	PRS	−0.04 (−0.10 to 0.02)	0.194
	VBS	−0.06 (−0.12 to −0.01)	0.019
	Interaction		0.359
**Odds of dementia**		**OR (95%CI)**	
Dementia	PRS	1.56 (1.17–2.09)	0.003
	VBS	1.38 (1.05–1.81)	0.018
	Additive interaction⁎⁎		0.460
	Multiplicative interaction		0.563

⁎ The polygenic risk score (PRS) for Alzheimer's disease is analyzed in increments of 1 standard deviation, whereas vascular burden score (VBS) is analyzed in 1-point increments (range 0–5).

⁎⁎ Based on relative excess risk due to interaction (RERI: 0.15, 95%CI: −0.25 to 0.55). The data are derived from multivariable models including age, sex, years of education, VBS, dementia PRS, and the first two ancestry principal components.

**Fig. 2:**
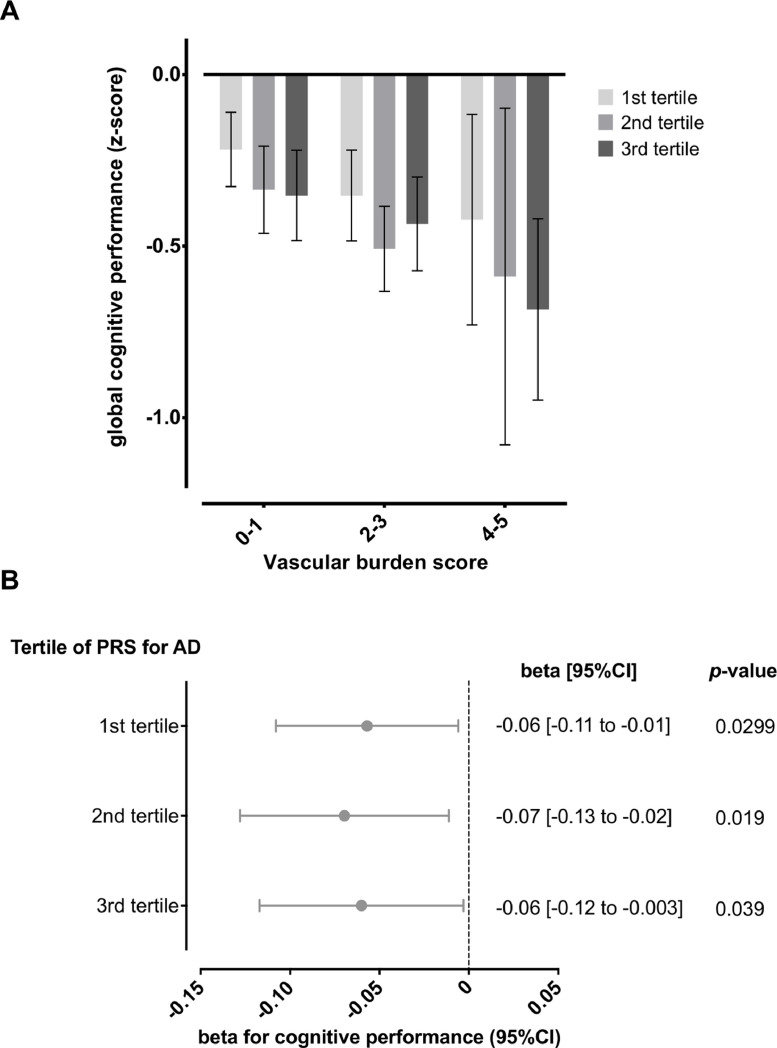
Associations of the vascular burden score with cognitive performance across tertiles of the polygenic risk score (PRS) for Alzheimer's disease. (A) Global cognitive performance across tertiles of the PRS for Alzheimer's disease by vascular burden score. (B) Results from linear regression regarding the effects of vascular burden score (1-point increment) across tertiles of the PRS for Alzheimer's disease. Results for panel B are derived from multivariable models including age, sex, years of education, vascular burden score (VBS), and the first two ancestry principal components.

Participants scoring higher in VBS showed worse global cognitive performance consistently across the three tertiles of the PRS for Alzheimer's disease (Fig. 2**A**). Indeed, in multivariable analyses, VBS showed consistent associations with worse global cognitive performance across all tertiles of the PRS for Alzheimer's disease (Fig. 2**B**). Again, similar associations were observed across all cognitive domains (**Supplementary Fig. 2**).

## Discussion

In this population-based study of 1172 community-dwelling individuals in Greece, both a higher VBS and a higher PRS for Alzheimer's disease were additively and independently of each other associated with worse global cognitive performance, worse domain-specific cognitive performance and higher odds of dementia. Across tertiles of the PRS for Alzheimer's disease, a higher VBS was equally associated with worse global cognitive performance. Even among individuals at highest genetic risk for dementia, VBS was still significantly associated with worse cognitive performance.

These data provide additional support to the notion of focusing on the modification of vascular risk factors and prevention of vascular disease in order to decrease the rates of dementia [50,51]. While several previous studies have provided evidence that vascular risk factors associate with the risk of dementia and cognitive impairment, it remained unclear if this risk is independent of the individual background genetic risk for dementia. Here, we show that there is no evidence of interaction between vascular burden and genetic dementia risk when it comes to their associations with cognitive performance and dementia. Furthermore, we show that even among individuals with a high baseline genetic risk for dementia, their vascular burden is still independently associated with cognitive performance. These results could serve as a basis for future *post hoc* analyses of clinical trials testing whether vascular risk factor modification, such as blood pressure lowering [52], could offset risk for cognitive decline even among individual at high genetic risk for dementia.

Our findings confirm and extend previous studies showing that modifiable risk factors can contribute to the risk of dementia independently of the individual genetic risk score for dementia. A study of 196,383 individuals in the population-based UK Biobank showed that a healthy lifestyle profile, as indicated by no current smoking, regular physical activity, healthy diet, and moderate alcohol consumption, was associated with a lower risk of dementia even among individuals at the highest genetic risk quantile for dementia [53]. Furthermore, among 1211 individuals of the Framingham Heart Study, low cardiovascular health index [54], was associated with a lower risk of incident dementia independently of a genetic risk score for dementia [55]. In the current study, we expand these findings beyond the hard outcome of dementia to performance across the entire spectrum of cognitive function. Furthermore, we use a burden index that beyond vascular risk factors, also incorporates information on presence of vascular disease including history of heart disease and cerebrovascular disease.

Several strengths of the current study should be noted. First, this is a population-based study representative of the general elderly population of community-dwelling individuals. Second, the extensive cognitive testing allowed for multiple layers of analyses with regards to cognitive performance. Third, a highly structured approach was followed for determining diagnoses of dementia involving a consensus meeting of neurologists and neuropsychologists on the basis of a detailed cognitive and functional assessment and inspection of all medical records of the participants.

This study has several limitations. First, the prevalence of dementia was relatively low in the overall sample (4%), probably reflecting an underrepresentation of individuals at high risk for dementia in the examined population. This may influence the external validity of our findings or add collider bias in the reported associations if the genetic score or the vascular risk factors also influenced participation in the study. Second, our analyses represent cross-sectional associations and cannot be used to establish causal associations between vascular burden and cognition, as reverse causation cannot be excluded. Third, unlike the genetic risk score for dementia, individuals were not randomly assigned to the vascular burden score and its associations with cognition could be biased by confounding. Fourth, this study is restricted to individuals of European ancestry and the results may thus not be generalizable to other populations. Fifth, several of our analyses have been limited by a relatively small sample size leading to uncertainty in many of the estimates, especially those referring to odds of dementia. Sixth, despite a rigorous evaluation of all individuals by neurologists and examination of pharmacotherapy and other background medical records (if available by participants), information for the vascular burden score was to a large extent based on self-report.

## Conclusions

In conclusion, in a community-based sample, vascular risk and genetic risk associate with cognitive performance and odds of dementia additively and independently of each other. Even among individuals at high genetic risk for dementia, a low vascular burden is associated with better cognitive performance. Whether targeting vascular risk factors could offset a high genetic risk score for dementia should be further explored in future studies.

## Sources of funding

This work has been supported by the following grants: IIRG-09–133,014 from the Alzheimer's Association, 189 10,276/8/9/2011 from the NSRF-EU program Excellence Grant (ARISTEIA), which is co-funded by the European Social Fund and Greek National resources, and ΔΥ2β/οικ.51657/14.4.2009 from the Ministry for Health and Social Solidarity (Greece). MG acknowledges support in form of a Walter-Benjamin fellowship from DFG (GZ: GE 3461/1–1) and from the FöFoLe program of LMU Munich (Reg.-Nr. 1120).

## Declaration of Competing Interest

Nothing to report.

## Appendix Supplementary materials

Image, application 1

## References

[1] Livingston G., Sommerlad A., Orgeta V., Costafreda S.G., Huntley J., Ames D. (2017). Dementia prevention, intervention, and care. Lancet.

[2] GBD Dementia Collaborators (2019). Global, regional, and national burden of Alzheimer's disease and other dementias, 1990-2016: a systematic analysis for the global burden of disease study 2016. Lancet Neurol..

[3] Pase M.P., Beiser A., Enserro D., Xanthakis V., Aparicio H., Satizabal C.L. (2016). Association of ideal cardiovascular health with vascular brain injury and incident dementia. Stroke.

[4] Hessler J.B., Ander K.H., Bronner M., Etgen T., Forstl H., Poppert H. (2016). Predicting dementia in primary care patients with a cardiovascular health metric: a prospective population-based study. BMC Neurol..

[5] Samieri C., Perier M.C., Gaye B., Proust-Lima C., Helmer C., Dartigues J.F. (2018). Association of cardiovascular health level in older age with cognitive decline and incident dementia. JAMA.

[6] Sabia S., Fayosse A., Dumurgier J., Schnitzler A., Empana J.P., Ebmeier K.P. (2019). Association of ideal cardiovascular health at age 50 with incidence of dementia: 25 year follow-up of Whitehall II cohort study. BMJ.

[7] Gardener H., Wright C.B., Dong C., Cheung K., DeRosa J., Nannery M. (2016). Ideal cardiovascular health and cognitive aging in the Northern Manhattan study. J. Am. Heart Assoc..

[8] Gottesman R.F., Schneider A.L., Zhou Y., Coresh J., Green E., Gupta N. (2017). Association between midlife vascular risk factors and estimated brain amyloid deposition. JAMA.

[9] Han J.W., Maillard P., Harvey D., Fletcher E., Martinez O., Johnson D.K. (2020). Association of vascular brain injury, neurodegeneration, amyloid, and cognitive trajectory. Neurology.

[10] Knopman D.S., Mosley T.H., Catellier D.J., Sharrett A.R. (2005). Atherosclerosis risk in communities S. cardiovascular risk factors and cerebral atrophy in a middle-aged cohort. Neurology.

[11] Gorelick P.B., Furie K.L., Iadecola C., Smith E.E., Waddy S.P., Lloyd-Jones D.M. (2017). Defining optimal brain health in adults: a presidential advisory from the American heart association/American stroke association. Stroke.

[12] Jansen I.E., Savage J.E., Watanabe K., Bryois J., Williams D.M., Steinberg S. (2019). Genome-wide meta-analysis identifies new loci and functional pathways influencing Alzheimer's disease risk. Nat. Genet..

[13] Kunkle B.W., Grenier-Boley B., Sims R., Bis J.C., Damotte V., Naj A.C. (2019). Genetic meta-analysis of diagnosed Alzheimer's disease identifies new risk loci and implicates Abeta, tau, immunity and lipid processing. Nat. Genet..

[14] Choi S.W., Mak T.S., O'Reilly P.F. (2020). Tutorial: a guide to performing polygenic risk score analyses. Nat. Protoc..

[15] Adams H.H., de Bruijn R.F., Hofman A., Uitterlinden A.G., van Duijn C.M., Vernooij M.W. (2015). Genetic risk of neurodegenerative diseases is associated with mild cognitive impairment and conversion to dementia. Alzheimers Dement.

[16] Chaudhury S., Brookes K.J., Patel T., Fallows A., Guetta-Baranes T., Turton J.C. (2019). Alzheimer's disease polygenic risk score as a predictor of conversion from mild-cognitive impairment. Transl. Psychiatry.

[17] Anastasiou C.A., Yannakoulia M., Kosmidis M.H., Dardiotis E., Hadjigeorgiou G.M., Sakka P. (2017). Mediterranean diet and cognitive health: initial results from the hellenic longitudinal investigation of ageing and diet. PLoS One.

[18] Dardiotis E., Kosmidis M.H., Yannakoulia M., Hadjigeorgiou G.M., Scarmeas N. (2014). The Hellenic Longitudinal Investigation of Aging and Diet (HELIAD): rationale, study design, and cohort description. Neuroepidemiology.

[19] Kosmidis M.H., Vlachos G.S., Anastasiou C.A., Yannakoulia M., Dardiotis E., Hadjigeorgiou G. (2018). Dementia prevalence in Greece: the Hellenic Longitudinal Investigation of Aging and Diet (HELIAD). Alzheimer Dis. Assoc. Disord..

[20] Ntanasi E., Yannakoulia M., Mourtzi N., Vlachos G.S., Kosmidis M.H., Anastasiou C.A. (2020). Prevalence and risk factors of frailty in a community-dwelling population: the HELIAD study. J. Aging Health.

[21] Tsapanou A., Gu Y., O'Shea D.M., Yannakoulia M., Kosmidis M., Dardiotis E. (2017). Sleep quality and duration in relation to memory in the elderly: initial results from the Hellenic Longitudinal Investigation of Aging and Diet. Neurobiol. Learn. Mem..

[22] Folstein M.F., Folstein S.E., McHugh P.R. (1975). "Mini-mental state". A practical method for grading the cognitive state of patients for the clinician. J. Psychiatr. Res..

[23] Lezak M.D. (2004).

[24] Vlahou C.H., Kosmidis M.H., Dardagani A., Tsotsi S., Giannakou M., Giazkoulidou A. (2013). Development of the Greek verbal learning test: reliability, construct validity, and normative standards. Arch. Clin. Neuropsychol..

[25] Kosmidis M.H., Vlahou C.H., Panagiotaki P., Kiosseoglou G. (2004). The verbal fluency task in the Greek population: normative data, and clustering and switching strategies. J. Int. Neuropsychol. Soc..

[26] Tsapkini K., Vlahou C.H., Potagas C. (2010). Adaptation and validation of standardized aphasia tests in different languages: lessons from the boston diagnostic aphasia examination - short form in Greek. Behav. Neurol..

[27] Benton A.L., Sivan A.B., Hamsher K., Varney N.R., Spreen O. (1994).

[28] Kosmidis M.H., Tsotsi S., Karambela O., Takou E., Vlahou C.H. (2010). Cultural factors influencing performance on visuoperceptual neuropsychological tasks. Behav. Neurol..

[29] Lezak M.D., Howieson D.B., Loring D.W. (2004).

[30] Bozikas V.P., Giazkoulidou A., Hatzigeorgiadou M., Karavatos A., Kosmidis M.H. (2008). Do age and education contribute to performance on the clock drawing test? Normative data for the Greek population. J. Clin. Exp. Neuropsychol..

[31] Vlahou C.H., Kosmidis M.H. (2002). The Greek trail making test: preliminary norms for clinical and research use. Psychol. J. Hell. Psychol. Soc..

[32] Giaglis G., Kyriazidou S., Paraskevopoulou E., Tascos N., Kosmidis M. (2010). Evaluating premorbid level: preliminary findings regarding the vulnerability of scores on cognitive measures in patients with MS [abstract]. J. Int. Neuropsychol. Soc..

[33] Bougea A., Maraki M.I., Yannakoulia M., Stamelou M., Xiromerisiou G., Kosmidis M.H. (2019). Higher probability of prodromal Parkinson disease is related to lower cognitive performance. Neurology.

[34] American Psychiatric Association (2000). Diagnostic and Statistical Manual of Mental Disorders (4th ed., text rev.).

[35] Blessed G., Tomlinson B.E., Roth M. (1968). The association between quantitative measures of dementia and of senile change in the cerebral grey matter of elderly subjects. Br. J. Psychiatry.

[36] Lawton M.P., Brody E.M. (1969). Assessment of older people: self-maintaining and instrumental activities of daily living. Gerontologist.

[37] Vlachos G.S., Kosmidis M.H., Yannakoulia M., Dardiotis E., Hadjigeorgiou G., Sakka P. (2020). Prevalence of mild cognitive impairment in the elderly population in Greece: results from the HELIAD study. Alzheimer Dis. Assoc. Disord..

[38] DeCarli C., Villeneuve S., Maillard P., Harvey D., Singh B., Carmichael O. (2019). Vascular burden score impacts cognition independent of amyloid PET and MRI measures of Alzheimer's disease and vascular brain injury. J. Alzheimers Dis..

[39] Toole J.F., Lefkowitz D.S., Chambless L.E., Wijnberg L., Paton C.C., Heiss G. (1996). Self-reported transient ischemic attack and stroke symptoms: methods and baseline prevalence. The ARIC study, 1987-1989. Am. J. Epidemiol..

[40] Bellenguez C., Küçükali F., Jansen I., Andrade V., Moreno-Grau S., Amin N., et al. New insights on the genetic etiology of Alzheimer's and related dementia. medRxiv 2020:2020.2010.2001.20200659.

[41] Grove M.L., Yu B., Cochran B.J., Haritunians T., Bis J.C., Taylor K.D. (2013). Best practices and joint calling of the HumanExome BeadChip: the CHARGE consortium. PLoS One.

[42] Purcell S., Neale B., Todd-Brown K., Thomas L., Ferreira M.A., Bender D. (2007). PLINK: a tool set for whole-genome association and population-based linkage analyses. Am. J. Hum. Genet..

[43] Abraham G., Qiu Y., Inouye M. (2017). FlashPCA2: principal component analysis of Biobank-scale genotype datasets. Bioinformatics.

[44] McCarthy S., Das S., Kretzschmar W., Delaneau O., Wood A.R., Teumer A. (2016). A reference panel of 64,976 haplotypes for genotype imputation. Nat. Genet..

[45] Karczewski K.J., Francioli L.C., Tiao G., Cummings B.B., Alfoldi J., Wang Q. (2020). The mutational constraint spectrum quantified from variation in 141,456 humans. Nature.

[46] Marchini J., Howie B., Myers S., McVean G., Donnelly P. (2007). A new multipoint method for genome-wide association studies by imputation of genotypes. Nat. Genet..

[47] Das S., Forer L., Schonherr S., Sidore C., Locke A.E., Kwong A. (2016). Next-generation genotype imputation service and methods. Nat. Genet..

[48] Loh P.R., Danecek P., Palamara P.F., Fuchsberger C., Reshef Y.A., Finucane H.K. (2016). Reference-based phasing using the haplotype reference consortium panel. Nat. Genet..

[49] Purcell S.M., Wray N.R., Stone J.L., Visscher P.M., O'Donovan M.C., International Schizophrenia Consortium (2009). Common polygenic variation contributes to risk of schizophrenia and bipolar disorder. Nature.

[50] Hachinski V., Einhaupl K., Ganten D., Alladi S., Brayne C., Stephan B.C.M. (2019). Preventing dementia by preventing stroke: the Berlin Manifesto. Alzheimers Dement.

[51] Pan Y., Li H., Wardlaw J.M., Wang Y. (2020). A new dawn of preventing dementia by preventing cerebrovascular diseases. BMJ.

[52] Hughes D., Judge C., Murphy R., Loughlin E., Costello M., Whiteley W. (2020). Association of blood pressure lowering with incident dementia or cognitive impairment: a systematic review and meta-analysis. JAMA.

[53] Lourida I., Hannon E., Littlejohns T.J., Langa K.M., Hypponen E., Kuzma E. (2019). Association of lifestyle and genetic risk with incidence of dementia. JAMA.

[54] Lloyd-Jones D.M., Hong Y., Labarthe D., Mozaffarian D., Appel L.J., Van Horn L. (2010). Defining and setting national goals for cardiovascular health promotion and disease reduction: the American Heart association's strategic impact goal through 2020 and beyond. Circulation.

[55] Peloso G.M., Beiser A.S., Satizabal C.L., Xanthakis V., Vasan R.S., Pase M.P. (2020). Cardiovascular health, genetic risk, and risk of dementia in the Framingham heart study. Neurology.

