# Supplementary material for: Vascular burden and genetic risk in association with cognitive performance and dementia in a population-based study
Source: Cereb Circ Cogn Behav. 2022 May 5;3:100145. doi: 10.1016/j.cccb.2022.100145 (PMC9616333; doi:10.1016/j.cccb.2022.100145)
Supplement: Supplementary file 1 [file mmc1.docx]

**SUPPLEMENTARY MATERIAL**

**Supplementary Table 1.** Genetic variants included in the polygenic risk score calculations for Alzheimer’s disease, derived from the study by Jansen *et al.* (2019) (*12*).

| **SNP** | **uniqID.a1a2** | **CHR** | **BP (hg19)** | **A1** | **A2** | **EAF** | **BETA** | **SE** |
| --- | --- | --- | --- | --- | --- | --- | --- | --- |
| rs4575098 | 1:161155392_A_G | 1 | 161155392 | A | G | 0.228 | 0.016 | 0.0026 |
| rs2093760 | 1:207786828_A_G | 1 | 207786828 | A | G | 0.192 | 0.024 | 0.0027 |
| rs4663105 | 2:127891427_C_A | 2 | 127891427 | C | A | 0.411 | 0.031 | 0.0022 |
| rs10933431 | 2:233981912_G_C | 2 | 233981912 | G | C | 0.244 | -0.015 | 0.0025 |
| rs6448453 | 4:11026028_A_G | 4 | 11026028 | A | G | 0.262 | 0.015 | 0.0025 |
| rs9381563 | 6:47432637_C_T | 6 | 47432637 | C | T | 0.356 | 0.014 | 0.0023 |
| rs1859788 | 7:99971834_A_G | 7 | 99971834 | A | G | 0.324 | -0.018 | 0.0023 |
| rs7810606 | 7:143108158_T_C | 7 | 143108158 | T | C | 0.485 | -0.015 | 0.0022 |
| rs4236673 | 8:27464929_A_G | 8 | 27464929 | A | G | 0.377 | -0.020 | 0.0022 |
| rs11257238 | 10:11717397_C_T | 10 | 11717397 | C | T | 0.361 | 0.013 | 0.0023 |
| rs2081545 | 11:59958380_A_C | 11 | 59958380 | A | C | 0.382 | -0.018 | 0.0022 |
| rs867611 | 11:85776544_G_A | 11 | 85776544 | G | A | 0.317 | -0.020 | 0.0023 |
| rs11218343 | 11:121435587_C_T | 11 | 121435587 | C | T | 0.044 | -0.036 | 0.0053 |
| rs12590654 | 14:92938855_A_G | 14 | 92938855 | A | G | 0.336 | -0.015 | 0.0023 |
| rs442495 | 15:59022615_C_T | 15 | 59022615 | C | T | 0.354 | -0.014 | 0.0023 |
| rs117618017 | 15:63569902_T_C | 15 | 63569902 | T | C | 0.125 | 0.018 | 0.0033 |
| rs59735493 | 16:31133100_A_G | 16 | 31133100 | A | G | 0.298 | -0.013 | 0.0024 |
| rs113260531 | 17:5138980_A_G | 17 | 5138980 | A | G | 0.126 | 0.020 | 0.0033 |
| rs28394864 | 17:47450775_A_G | 17 | 47450775 | A | G | 0.453 | 0.012 | 0.0022 |
| rs111278892 | 19:1039323_G_C | 19 | 1039323 | G | C | 0.147 | 0.020 | 0.0031 |
| rs41289512 | 19:45351516_G_C | 19 | 45351516 | G | C | 0.036 | 0.206 | 0.0058 |
| rs3865444 | 19:51727962_A_C | 19 | 51727962 | A | C | 0.299 | -0.014 | 0.0024 |
| rs6014724 | 20:54998544_G_A | 20 | 54998544 | G | A | 0.095 | -0.023 | 0.0037 |

SNP, single nucleotide polymorphism; CHR, chromosome; BP, base-pair position according to hg19; A1, effect allele; A2, other allele; EAF, effect allele frequency; BETA, log-odds ratio of the association of the effect allele with Alzheimer’s disease; SE, standard error.

**Supplementary Figure 1.** Polygenic risk score (PRS) for Alzheimer’s disease across categories of the vascular burden score.

**Supplementary Figure 2.** Associations between the vascular burden score with cognitive performance in different domains across tertiles of the polygenic risk score for Alzheimer’s disease.

Results for are derived from multivariable models including age, sex, years of education, vascular burden score (1-point increment), and the first two ancestry principal components.
